# Screening and Caring for Older Adults Affected by Sexual or Other Types of Violence: A Pilot Study at Three Belgian Geriatric Departments

**DOI:** 10.3390/healthcare14010016

**Published:** 2025-12-20

**Authors:** Charlotte Boven, Anne Nobels, Nicolas Berg, Nele Van Den Noortgate, Nathalie Courtens, Ines Keygnaert

**Affiliations:** 1 International Centre for Reproductive Health (ICRH) & VIORESC, Ghent University, 9000 Ghent, Belgium; ines.keygnaert@ugent.be; 2 Women’s Clinic, Ghent University Hospital, 9000 Ghent, Belgium; 3 Department of Geriatric Medicine, Ghent University Hospital, 9000 Ghent, Belgium; anne.nobels@uzgent.be (A.N.); nele.vandennoortgate@uzgent.be (N.V.D.N.); 4 Department of Psychiatry, Ghent University Hospital, 9000 Ghent, Belgium; 5 Department of Geriatric Medicine, University Hospital Liège, 4000 Liège, Belgium; berg@docteurberg.be; 6 Department of Internal Medicine and Pediatrics, Ghent University, 9000 Ghent, Belgium; 7 Sexual Assault Care Centre (SACC), AZ Delta, 8800 Roeselare, Belgium; nathalie.courtens@azdelta.be; 8 Department of Public Health and Primary Care, Ghent University, 9000 Ghent, Belgium

**Keywords:** elder abuse, geriatric care, hospitals, intervention, older adults, screening

## Abstract

**Highlights:**

**What are the main findings?**

- The guide was pilot tested in three geriatric departments with a total of 104 older adults. Older adults are willing to disclose experiences of violence to healthcare providers.
- A comprehensive trauma-informed guide was developed, including screening questions and referral pathways, to identify all forms of violence experienced by older adults, regardless of when the violence occurred
- This guidance supports healthcare providers in engaging in these conversations; however, additional education and training are necessary.

**What is the implication of the main finding?**

- Older adults do not find the questions intrusive and appreciate being listened to.
- Healthcare providers should refrain from falling into the trap of problem-solving.

**Abstract:**

**Background/Objectives:** Violence against older adults is a rising public health issue. Though older adults may not openly disclose such experiences, they are often willing to discuss them when given the opportunity. Healthcare providers in hospital settings can play a crucial role in the early identification and care. However, effective screening and response require comprehensive guidance. **Methods:** A pilot, multicentric feasibility study with a single-group intervention was implemented at three Belgian geriatric departments. The aim was to assess the feasibility and acceptability of a new guide for identifying older adults (≥75 years), without major cognitive deficits, who have experienced violence, in order to subsequently provide them with adequate care. Admitted older adults were screened using the guidance, and healthcare providers who conducted the screenings completed questionnaires to evaluate their feasibility and acceptability. The Trial is registered in Clinicaltrials.gov [NCT06780540]. **Results:** A total of 104 admitted older adults (mean age: 83 years) were recruited across two Dutch-speaking and one French-speaking hospital in Belgium. One in five participants (20.2%) disclosed experiences of violence, either recent or throughout their lives. Healthcare providers (*n* = 12) positively evaluated the guidance, suggesting improvements in question formulation, protocol adaptability, and the need for further training. **Conclusions:** This guidance is feasible, acceptable, and holds potential for improving disclosure rates. To ensure the provision of appropriate and equitable care, it is essential to first equip healthcare providers with education and training on this topic. Future interventional research is required to implement the guide on a larger scale and to measure health-related outcomes.

## 1. Introduction

Violence in older adults is a growing public health and human rights concern [1] that affects many individuals worldwide [2,3,4]. Experiences of violence can severely impact older adults’ health, both in the short and long term [5,6], including physical and psychological morbidity [7,8], higher rates of suicidal thoughts [9], and premature mortality [10]. It can occur in the following contexts: homes, care institutions, public spaces, and the Internet [3,4]. Reliable prevalence data remain scarce due to factors such as inconsistent data collection [4,11], varying study methodologies [3], and underreporting [12]. The meta-analysis of Yon, Mikton, and Gassoumis [13] estimated that globally 15.7% of adults aged 60 and older experienced violence in community settings over the past year, but the actual magnitude is likely higher. Studies suggest that only one in 24 cases is reported [12]. As the global population ages, these numbers are expected to increase.

Violence takes various forms: physical, psychological, or sexual violence, material exploitation or financial violence, and neglect [14]. It may occur as a single incident, repeated incidents over a lifetime, or multiple forms simultaneously [15]. Violence in older adults, particularly sexual violence, remains underrecognized in research, policy, and clinical practice [2]. Older adults are often perceived by the public and healthcare providers as unlikely targets of sexual violence [16,17], which contributes to research gaps, a lack of evidence-based interventions, and the exclusion of older adults from policies, guidelines, and prevention programs [2]. Despite common misconceptions, sexual violence in older adults is relatively prevalent. In Belgium, a study found a 44.2% lifetime prevalence and 8.4% prevalence in the past year. Additionally, the recent establishment of Belgian Sexual Assault Care Centres (SACCs) in 2017 underscores the need to educate healthcare providers, as holistic care improves recovery outcomes and reduces the risk of revictimization [18].

The belief that older adults are unlikely victims of violence reflects ageism, which is understood as the stereotyping, discrimination, or holding of prejudices against a person based on their age. Ageism includes attitudes, behaviors, and policies that disadvantage or exclude older adults [19]. A European study found that one in four (24.9%) individuals aged 65 and older reported experiencing ageism [20]. This number is concerning, as ageism contributes to poorer health outcomes among older adults [21] and discourages professional help-seeking as older victims may feel undeserving of care [22,23]. Older adults, particularly those experiencing sexual violence, are often reluctant to disclose to healthcare professionals [17,22,23,24]. As a result, violence often remains unnoticed [3,4] and access to professional care is limited [22]. Previous research [8,25] has shown a clear link between lifetime sexual victimization and mental health issues in old age, and has indicated that older victims of sexual violence experience significant distrust toward others, which may influence how they interact with healthcare workers. Therefore, screening for both recent and non-recent (sexual) violence is an important intervention to improve the care of older victims of (sexual) violence. Healthcare providers are obliged to protect patients from continued harm, but often fail to recognize violence in older patients [26].

To address and prevent violence, clear protocols, adequate staffing and training, and a supportive environment are essential [27]. Most healthcare providers lack training and tools to identify and manage cases of sexual violence [17,28,29] or other forms of violence [30]. The high prevalence of violence, barriers to disclosure, and associated health consequences underscore the urgent need for increased awareness, improved screening, and enhanced care in hospital settings. Because forms of violence often co-occur, a broader screening approach is necessary [31]. Additionally, the potential long-term impact of violence and the increased risk of revictimization [32,33] reinforce the importance of assessing all forms of violence, regardless of when they occurred.

A number of screening tools exist, which cover all types of abuse or a specific form. A review by Gallione, Dal Molin, Cristina [34] shows four comprehensive detection tools: Vulnerability Abuse Screening Scale (VASS), Hwalek-Sengstock Elder Abuse Screening Test (H-S/EAST), Elderly Indicators of Abuse or Indicators of Abuse (E-IOA or IOA), and the Elder Abuse Suspicion Index (EASI). For older adults in Belgium, there is another screening tool available: Risk on Elder Abuse and Mistreatment-Instrument (REAMI or RITI), but this tool only exists in Dutch [11]. Currently, no screening tool specifically addresses sexual violence in older adults. Existing instruments, such as the Elder Abuse Suspicion Index, only tangentially address it. Literature suggests adapting these tools [35] and establishing effective care procedures to improve responses to disclosures [4,11,22].

This paper introduces a new guidance tool that helps healthcare providers identify older adults (without cognitive problems) who have experienced violence and ensure they receive appropriate care after disclosure. The feasibility of the selected population (people of ≥75 years and/or highly care dependent without cognitive problems) and setting (geriatric care), but also the feasibility and acceptability of guidance itself (the screening tool and care and referral pathways) was explored in a pilot, multicentric feasibility study in which a single-group intervention was implemented in three geriatric departments.

## 2. Materials and Methods

The guidance is part of the larger Operation Alert project, which is commissioned by the Belgian Federal Public Service of Public Health, and implemented by Ghent University Hospital (UZGent), Ghent University (UGent-ICRH/VIORESC), and an NGO on female genital mutilation (GAMS vzw). Operation Alert is a capacity-building project for all staff members in Belgian hospitals, boosting their capacities to detect signals of domestic and sexual violence and to improve their care for victims through e-learning tracks and hands-on care and referral pathways.

The guidance aims to identify older adults who have experienced sexual or other forms of violence (physical, including neglect, psychological, socio-economic, and economic violence), either recently (<one month) or in the past (>one month), in order to provide appropriate care upon positive screening. Definitions of the different types of violence are listed in Appendix A.

The aim of the study was to pilot test the guide, more specifically the screening tool and referral pathways, to assess the feasibility and acceptability of the selected population, setting, and guidance. Its focus was on improving holistic, trauma-sensitive care to older victims of violence, and not on reporting population-based prevalences or factors associated with violence. Prevalences of lifetime sexual violence in older adults are published elsewhere [17].

### 2.1. Participants

#### 2.1.1. Intervention with Older Adults

Eligible participants were invited to receive the intervention. Participants were Dutch- or French-speaking individuals aged ≥75 or highly care-dependent, with reasonable cognitive functioning, admitted to the geriatric departments of AZ Delta, CHU de Liège, and UZ Gent between 16 December 2024 and 31 March 2025. The hospitals were selected based on their provision of acute geriatric care, their geographical distribution across Belgium, and their integration of a Sexual Assault Care Centre (SACC) where holistic, specialized care is provided to victims of acute sexual violence. Older adults admitted to one of the three participating geriatric departments were screened using the guidance, having first provided written informed consent. Patients were excluded if their health condition was too severe for study participation, worsened between informed consent and screening, or if cognitive issues (e.g., delirium) were noticed before or during the screening.

The age threshold of 75 years old or above and the level of care dependency align with the admission criteria for Belgian geriatric departments. Admissions include both acute care units, where patients stay for multiple days, and day clinics, where patients receive diagnoses (e.g., cognitive impairment) or treatments (e.g., intravenous therapy) within a single day. It is important that screening is conducted in professional care settings, such as hospitals, since older adults generally use healthcare services more often. The geriatric department was considered an ideal setting due to its multidisciplinary team meetings and comprehensive geriatric assessments. While other settings, such as residential care, may provide more time to build trusting relationships and facilitate such conversations, it is important to screen for violence in all care settings. Older adults who experience violence from a caregiver in one care setting may feel safer disclosing it in a different care environment where they are not dependent on the perpetrator.

#### 2.1.2. Evaluation with Healthcare Providers

Participants were Dutch- or French-speaking healthcare providers working in the geriatric departments or Sexual Assault Referral Centers (SARC) of AZ Delta, CHU de Liège, and UZ Gent. We selected a variety of professional profiles to illustrate that the guide can be used across different roles. Because it is intended to be accessible to all healthcare providers working with older adults, we also wanted to assess whether involving different profiles was both feasible and acceptable. The healthcare providers in this study were a convenience sample of different profiles from participating settings. After having followed training, they implemented the guidance between 16 December 2024, and 31 March 2025. Healthcare providers, who carried out the screening, gave written consent and subsequently completed an evaluation on the guidance.

### 2.2. Study Materials

As mentioned in the background, several screening tools exist. However, the VASS and RITI are designed for use in home care, whereas the HS-EAST, EASI, and E-IO can be applied in a hospital setting. The IOA and E-IOA require a lengthy completion time of two to three hours, which may be burdensome for older adults with (acute) health problems admitted to the hospital. In contrast, the EASI can be administered in approximately two minutes [11]. However, it lacks behavior-specific questions (BSQ) related to sexual violence, which is an important limitation when working with older adults whose generational beliefs (e.g., about marital rape) may influence how they understand and report sexual violence. The BSQ uses specific, concrete descriptions to frame incidents, rather than broad labels such as ‘rape’, thereby limiting ambiguity in interpretation. Participants who may not recognize their own experiences of sexual victimization or link the questions to their own experiences may not disclose their experiences of lifetime sexual violence [36]. The BSQs were based on a previous study (UN-MENAMAIS) on sexual violence in older adults [28], and aligned with the WHO definition of sexual violence, which includes various forms of sexual violence, such as sexual harassment without physical contact, sexual abuse involving physical contact without penetration, and (attempted) rape. Another motivation for developing the guide was that the aforementioned screening tools rely solely on dichotomous questions, whereas we aimed to include open questions as well. Findings from our focus groups and expert consultations highlighted the need for such open questions, as they help create a safe and compassionate environment that allows space for patients to share their lifetime experiences of violence and talk about their network and underlying dynamics.

The development of the guidance was performed through an iterative process of literature reviews, input from national and international experts, and focus group discussions before piloting was conducted. Detailed information on the research process and results is available in Appendix B. The guidance encompasses three core components: a screening protocol, care and referral pathways, and a theoretical foundation on sexual violence. All study materials are available in both French and Dutch, in accordance with Belgium’s bilingual context.

#### 2.2.1. Intervention with Older Adults

In addition to reviewing the guidance, healthcare providers completed a self-paced, evidence-based e-learning module developed for Operation Alert focused on the experiences of violence among older adults. SARC healthcare providers have extensive experience in talking about violence, and have followed the e-learning to learn more about violence in older adults. Those from the geriatric department have extensive experience in talking with older adults, and followed the e-learning to broaden their competencies on violence in older adults. Accessible between December 2024 and March 2025, the 45–60-min module addressed the dynamics of violence, including risk and protective factors, warning signs, and intervention strategies. It also supported professionals in initiating conversations about (suspected) violence with older adults, colleagues, and potentially abusive caregivers, while offering guidance on delivering holistic care to all involved.

The screening tool (see Appendix C) was integrated into routine geriatric assessments. Patients were informed through consent procedures that participation was voluntary, and they could decline or stop at any time. Screenings were conducted privately, without relatives present, in one or two phases by professionals from various backgrounds.

Providers had flexibility in administering the tool, including adapting the phrasing or sequence of questions. However, any modifications to the screening protocol or care pathways had to be documented in a brief interim questionnaire. This form also included sections for feedback on patient reactions and any issues with implementation, such as unclear items or missing referral contacts. If no changes or issues were observed, completion of the interim questionnaire was not required.

#### 2.2.2. Evaluation with Healthcare Providers

The feasibility and acceptability of the newly developed guidance [37] were evaluated by healthcare providers using an online questionnaire (Qualtrics) based on the theoretical framework of acceptability by Sekhon, Cartwright, and Francis [38], which assesses seven constructs: affective attitude, burden, ethicality, intervention coherence, opportunity costs, perceived effectiveness, and self-efficacy. Healthcare providers rated each construct on a five-point Likert scale with different response options. In addition to these closed-ended items, the questionnaire included open-ended questions, allowing healthcare providers to provide feedback on feasibility (e.g., required resources, implementation barriers, and suggestions for improvement). Older adults did not directly evaluate the feasibility or acceptability of the guidance, as relevant items were discreetly embedded in routine care (e.g., the geriatric assessment), making it difficult for patients to distinguish between the intervention and standard practice. Moreover, we wanted to limit the administrative burden associated with hospital admission, because some patients might not be able to complete the evaluation independently. They would need help from (potentially) abusive caregivers or from the researcher, which in the latter case could bias the results.

### 2.3. Ethics

The research protocol was approved by the Ethics Committee of Ghent University Hospital [ONZ-2024-0077]. Healthcare providers and older adults received verbal and written information about the study and gave their explicit and written consent to participate. The study was also registered in a publicly accessible database [NCT06780540] of the National Library of Medicine (NIH) and released on 13 January 2025.

The study was conducted in accordance with the WHO’s ethical and safety recommendations for conducting research on sexual violence. Participants received detailed information about the study in advance. Informed consent was obtained prior to participation, and participants could withdraw their consent at any time. They could also always pause or stop the conversation at any time. They were also informed that their participation or non-participation would not affect the care they received. Participating healthcare providers ensured that the screening was conducted in a safe, nonjudgmental environment, without other patients, relatives, or healthcare providers present. After the conversation, older adults were asked whether they wanted to receive any further support, and what type if they did. Informed consent also included contact information for both the geriatric department and the research team. The guide also included referral pathways depending on the nature and acuteness of the violence. The steps outlined for acute or non-acute violence were in accordance with Article 458 of the Belgian Criminal Code, and the flowchart was extensively reviewed by research and clinical experts in this field.

## 3. Results

### 3.1. Intervention with Older Adults (n = 104)

**Figure 1:**
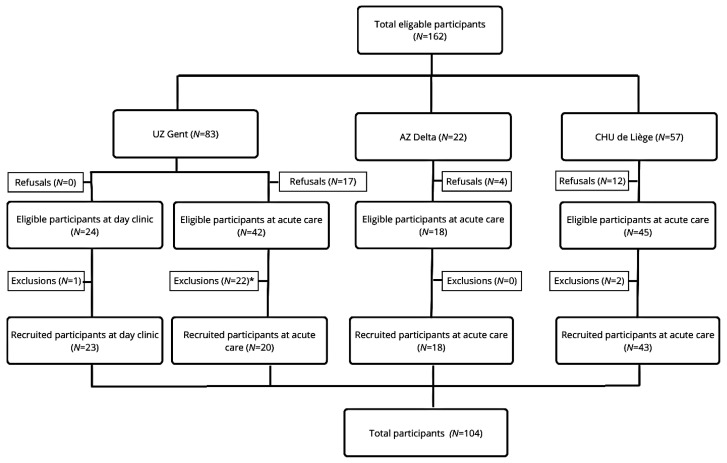
Flow chart of the multicentric interventional pilot study. Legend: * The exclusions at UZ Gent (acute care) were primarily due to the time lapse between obtaining informed consent and conducting the screening at the Acute Care Department of Geriatric Medicine. Ultimately, 22 patients could not be included due to various factors, such as deteriorating health, unexpected deaths, cognitive deficits or temporary confusion (e.g. delirium), or early hospital discharge. Consequently, there was a discrepancy between the number of signed informed consent forms (*N* = 42) and the number of patients who ultimately participated in the study (*N* = 20).

A total of 104 eligible participants were recruited from three different sites: CHU de Liège (*n* = 43), UZ Gent (*n* = 43), and AZ Delta (*n* = 18). Several patients refused to participate (*n* = 33) and/or were excluded from participation (*n* = 25). Some patients declined participation due to disinterest, ongoing treatments, providing only oral informed consent, but refusing to sign the paperwork, and some patients felt too ill or in too much pain to participate. A study flowchart with more information about the recruitment of participants on the different sites can be found in Figure 1.

Each hospital site faced specific challenges that complicated the recruitment process, including viral outbreaks, competing care priorities, and staff shortages or overburdened healthcare teams. Due to staffing constraints, the screening at AZ Delta was carried out by healthcare providers (nurses and psychologists) from the Sexual Assault Care Centre rather than the geriatric department. However, these healthcare providers took deliberate steps to build trust with patients before administering the screening questions.

Of the 104 participants included in the study, the majority were women (*n* = 62). Additionally, 45 participants lived with their partner. The mean age of the participants was 83 years [range: 75–102 years]. More information on the patients’ characteristics can be found in Table 1.

One in five participants (20.2%) disclosed experiences of violence, either recent or throughout their lives. The types of violence reported included economic violence (*n* = 7), of which six were burglaries, psychological violence (*n* = 6), sexual violence (*n* = 3), physical violence (*n* = 2), neglect (*n* = 2), and socio-economic violence (*n* = 1). A higher number of disclosures were reported by women (*n* = 13) than by men (*n* = 8), with this difference nearing statistical significance (Fisher’s Exact Test, *p* = 0.05).

### 3.2. Evaluation with Healthcare Providers (n = 12)

Twelve healthcare providers who implemented the screening with older adults at CHU de Liège, UZ Gent, and AZ Delta completed a questionnaire at the end of the intervention period. The questionnaire included both open- and closed-ended items rated on a five-point Likert scale (see Appendix D), and results indicated an overall positive evaluation of the guidance. Further details on the healthcare providers’ characteristics and their evaluation are presented in Table 2.

Responses to the closed-ended questions (see Table 2) highlighted several key strengths, such as its perceived positive contribution to the patient’s well-being, the patient’s acceptability of the screening protocol, healthcare providers’ confidence in using the protocol, and their recommendations for its use by others.

The open-ended questions revealed that healthcare providers believed it is important for the screening to be integrated into existing standards of care to avoid increasing their workload. They also emphasized that the guidance can raise awareness among healthcare providers, enhance their vigilance, and make conversations about violence more common. At the same time, they stated that implementing this approach will require a shared vision and regular ethical reflections within a team. The systematic incorporation of screening into routine practice, coupled with the initiative of healthcare providers, may increase the likelihood of disclosure by reducing barriers to reporting. Healthcare providers noted that the guidance helps patients to feel seen and heard; hence, their experiences of violence and the impact it has on them are acknowledged. They reported that patients responded very positively to the conversation and appreciated that healthcare providers took the time to listen to them. Older adults did not find the questions to be intrusive, but experienced them as ordinary. Disclosures provided the opportunity to offer tailored care, if desired by the older adult, which was often not the case. According to the healthcare providers, it was often sufficient for patients to be offered a sympathetic ear, rather than a formal intervention, emphasizing the need to refrain from falling into the trap of problem-solving.

The evaluation of the guidance also identified several areas for improvement. First, the phrasing of the screening questions needs to be adjusted to make them easier to understand. Additionally, there is a need for examples to clarify what constitutes different types of violence. Second, it was emphasized that the guidance should support more natural conversations with patients, rather than relying on a rigid screening protocol. Third, in addition to the screening protocol, further training and education are needed to help healthcare providers develop conversational skills that will enable them to engage with patients (e.g., trauma-sensitive care and adequate follow-up questions) and abusive caregivers (e.g., psycho education), but also adequately respond to disclosures of violence.

## 4. Discussion

Although several screening tools exist for detecting and supporting older adults affected by violence, there has been limited focus on sexual violence and non-recent experiences. This study led to the development of a comprehensive guidance designed to identify all forms of violence experienced by older adults, including sexual violence, regardless of when the violence occurred. The guidance is informed by trauma-informed care principles, which are crucial given the high likelihood of cumulative trauma in older populations, minimizing the risk of re-traumatization. The aim of the pilot study was to explore whether the guidance can be practically implemented on a larger scale within this specific setting (geriatric care) and population (people of ≥75 years and/or highly care dependent). The feedback from healthcare providers demonstrated its feasibility and acceptability among healthcare providers working in acute geriatric care settings. It was indicated that the guidance has the potential to enhance the identification and disclosure of sexual and other types of violence, ultimately facilitating timely and appropriate care. Healthcare providers also encountered challenges while screening, such as patients’ medical conditions (e.g., severe illness), early discharge, significant cognitive impairments, and temporary confusion (e.g., delirium).

A key strength of this study is the inclusion of a geriatric population with a mean age of 83 years, a demographic frequently underrepresented in research on violence. Whereas prior studies [39] have predominantly focused on younger, more active individuals aged 65 and older, this study specifically includes older adults characterized by higher levels of frailty and comorbidity. This inclusion enhances the clinical relevance of the findings and demonstrates that the guidance is applicable within a geriatric population, where advanced age and multimorbidity are common. Although the guidance proved feasible for cognitively intact older adults in an acute geriatric ward, its generalizability to other healthcare settings, such as different hospital departments, psychiatric units, primary care, or long-term residential facilities, remains to be established. Each setting presents distinct challenges related to patient profiles and healthcare provider roles, which can impact both implementation and effectiveness. A limitation of our study is the small sample size of the healthcare providers who conducted the screening and subsequently completed the questionnaire, which may have introduced bias. It is possible that the professional background (e.g., nurse versus psychologist) and level of experience in communicating with older adults may have influenced our findings.

Participants in our study underscored the importance of integrating screening protocols within existing care frameworks, notably the comprehensive geriatric assessment. However, such integrative assessments are not universally conducted across all healthcare environments, which may limit the guidance’s applicability. Moreover, cognitive impairment is prevalent in geriatric populations, yet current guidelines are primarily designed for those with sufficient cognitive capacity to engage in screening processes. Future research should investigate violence prevalence and characteristics among cognitively impaired individuals, including those with dementia, a known risk factor for victimization [40]. Further evaluation of the guidance through rigorous methodologies, such as cluster randomized controlled trials, is also warranted to assess its effectiveness compared to standard care. In this design, clusters begin in the control condition and are randomly assigned to sequences. They then cross over to the intervention condition at a predetermined time point until all clusters have received the intervention [41]. Overall, while this study focused on feasibility, acceptability, and identifying limitations, subsequent research should aim to confirm the guidance’s impact on initiating adequate support and ultimately reducing long-term mental health consequences for older adults affected by violence. It is also important to note that we encourage to gain input from older victims of violence. This will help to further reflect and substantiate the guide, as it is a living document that will need to be regularly updated in time (e.g., referral pathways to organizations and medical care, …).

In our study, one in five older adults disclosed having experienced violence, though the true prevalence is likely higher [12]. Social context significantly influences whether individuals choose to disclose such experiences or seek help [42]. Healthcare providers reported that older adults generally did not mind being asked about potential violence, highlighting the importance of creating a safe, supportive, and nonjudgmental environment to encourage disclosure [16,43,44]. Building trust, rapport, and practicing active listening are essential to facilitate openness [45]. However, research indicates that disclosure of sexual violence alone does not necessarily improve mental health outcomes. Instead, the response to disclosure plays a critical role [46]. Negative reactions, such as controlling behavior, dismissal, or differential treatment, are linked to worse psychological outcomes [47]. These findings emphasize the urgent need for training of healthcare providers aimed at reducing harmful social responses. Healthcare providers also noted that, in most cases, older adults simply wanted someone who listened to their story in a compassionate way. Many older adults do not always identify themselves as victims of sexual or other types of violence due to factors such as internalized ageism or a desire to protect family ties [16,23]. They may downplay their experiences by attributing behaviors to caregiver stress [43] or describing sexual violence as “just something that happened” [46]. Consequently, rigid screening protocols may be inadequate, as older adults might not recognize or label their experiences as violent. In these situations, healthcare providers can play a crucial role in helping older adults recognize their experiences as violent, which is an essential first step towards help-seeking behaviors [48]. The feedback from the pilot study informed the development of a broader and more flexible screening protocol (see Appendix E), designed to create space for older adults to speak freely. Individuals may be reluctant to disclose experiences of violence during initial screening, as they may need time and space to decide, and future research could explore whether readmitted patients might report such experiences at a later stage by implementing the guide for a longer period of time.

Finally, healthcare providers highlighted the critical need for comprehensive training and education to develop competencies necessary to address sexual and other forms of violence in older adults. Previous research shows that many healthcare professionals lack competencies in this area, largely due to limited educational opportunities [30,49]. Our findings, along with previous research, reinforce the urgency to invest in specialized training programs, which have been shown to enhance knowledge, increase the use of assessment tools, reduce reports of abusive actions by staff [50], and effectively reduce ageism [19]. Every healthcare provider working with potential victims of violence in hospital settings should be able to recognize signs of violence and provide basic support. In addition to the basic competences, we recommend that those who conduct the screening should also possess more advanced skills and knowledge (e.g., recognize ageist attitudes, understand cumulative trauma, navigate referral pathways). Therefore, the guide includes references to relevant international literature and e-learning modules that were specifically developed, as well as this guide, as part of a larger governmentally funded project called “Operatie Alert” (Operation Alert).

Additionally, healthcare providers emphasized the importance of collective responsibility and ongoing critical reflection within healthcare teams. Routine screening for violence can have a psychological impact on healthcare providers, as repeated exposure to traumatic accounts or direct encounters with violence may result in vicarious trauma, especially among those with personal histories related to the patients they treat [49]. Research estimates that between 40% and 85% of healthcare providers may experience vicarious trauma, compassion fatigue, or heightened trauma-related symptoms due to prolonged engagement with traumatized populations [51]. Therefore, it is equally important to incorporate guidance on managing vicarious trauma and promoting self-care in training programs.

## 5. Conclusions

The guidance developed for screening both recent and non-recent experiences of sexual and other forms of violence was found to be feasible and acceptable within a geriatric care setting. However, it is important to acknowledge that the pilot study included only a specific subset of the population, as individuals with major cognitive impairments and those who were too ill to participate were excluded. Additionally, healthcare providers reported that patients responded positively to the screening questions and did not find them intrusive. We recommend that future research conduct feasibility studies in other care contexts, such as additional hospital departments or primary care settings, and interventional studies with clear health-related outcome measures. Finally, we believe it would be valuable to gain direct input from older victims of violence to further reflect and substantiate the guide, as it is a living document that will need to be regularly updated.

## Figures and Tables

**Table 1:** Characteristics of the older adults participating in the pilot study (*n* = 104) with no missing data.

Characteristics Older Adults (*n* = 104)	
Gender	
Female	62 (59.6%)
Male	42 (40.4%)
Age categories	
75–80	40 (38.5%)
81–85	28 (26.9%)
86–90	24 (23.1%)
91–95	11 (10.6%)
101–105	1 (1%)
Mean age [range: 75–102 years]	83 years
Living conditions:	
Living together with partner/spouse	45 (43.3%)
Partner or spouse, but living apart	4 (3.8%)
No partner or spouse (alone)	37 (35.6%)
Other	16 (15.4%)

**Table 2:** The characteristics of the healthcare providers (*n* = 12) who conducted the pilot study, as well as their evaluations of the newly developed guidance. For analytical clarity and due to the small sample size, responses on the original five-point Likert scale were transformed into three categories. There is no missing data.

Healthcare Providers (*n* = 12)	
Characteristics of Healthcare Providers	
Gender	
Female	10 (83.3%)
Male	2 (16.7%)
Function	
Nurse	6 (50%)
Psychologist	3 (25%)
Occupational therapist	2 (16.7%)
Physician	1 (8.3%)
Years of work experience (mean) [range: 5–41 years]	20 years
**Evaluation of the guidance for older adults and sexual or other types of violence**	
To what extent do you consider the screening protocol a useful tool for detecting experiences of violence in your patients? (Median: 3)	
3: Useful or very useful	9 (75%)
2: No opinion	1 (8.3%)
1: Somewhat or not useful	2 (16.7%)
How likely are you to recommend the use of the screening protocol to your colleagues? (Median: 3)	
3: Rather or strongly recommend	10 (83.3%)
2: No opinion	2 (16.7%)
1: Rather not or not recommend	0
How easy was it to incorporate the screening protocol into the geriatric assessment? (Median: 3)	
3: Rather or very easy	10 (83.3%)
2: No opinion	1 (8.3%)
1: Rather or very difficult	1 (8.3%)
To what extent do you feel that carrying out the screening protocol was an effort for you? (Median: 3)	
3: Little to no effort	8 (66.7%)
2: No opinion	1 (8.3%)
1: Quite or a lot of effort	3 (25%)
To what extent did the screening protocol interfere with your other priorities in patient care? (Median: 3)	
3: Rather no or no interference	8 (66.7%)
2: No opinion	3 (25%)
1: Some or a significant interference	1 (8.3%)
If you had a patient with an experience of violence, did the care and referral processes adequately support you in providing appropriate care? (Median: 2)	
3: Rather or very well supported	1 (8.3%)
2: No opinion	10 (83.3%)
1: Minimally or not supported at all	1 (8.3%)
To what extent do you think the guidelines (screening protocol and care and referral pathways) have positively contributed to your patient’s well-being? (Median: 3)	
3: Rather or very positive contribution	10 (83.3%)
2: No opinion	2 (16.7%)
1: Rather negative contribution	0
To what extent do you think patients found it acceptable to receive the screening questions during the geriatric assessment? (Median: 3)	
3: Rather or very acceptable	10 (83.3%)
2: No opinion	2 (16.7%)
1: Rather or very unacceptable	0
To what extent do you think your patients found the content of the screening questions acceptable? (Median: 3)	
3: Rather or very acceptable	12 (100%)
2: No opinion	0
1: Rather or very unacceptable	0
To what extent do you think your patients found the formulation of the screening questions acceptable? (Median: 3)	
3: Rather or very acceptable	8 (66.7%)
2: No opinion	3 (25%)
1: Rather or very unacceptable	1 (8.3%)
How confident did you feel when using the screening protocol? (Median: 3)	
3: Rather or very confident	11 (91.7%)
2: No opinion	1 (8.3%)
1: Rather or not confident at all	0
To what extent do you think the screening protocol gave you enough knowledge and skills to work with? (Median: 2.5)	
3: Provided knowledge and skills to some extent or a lot	6 (50%)
2: No opinion	3 (25%)
1: Provided little or no knowledge and skills	3 (25%)
If you had a patient who experienced violence, do you feel the care and referral processes provided you with adequate knowledge and skills to take action? (Median: 2)	
3: Provided knowledge and skills to some extent or a lot	2 (16.7%)
2: No opinion	9 (75%)
1: Provided little or no knowledge and skills	1 (8.3%)

## Data Availability

The datasets presented in this article are not readily available due to the inclusion of sensitive or confidential information. Requests to access the datasets should be directed to charlotte.boven@uzgent.be.

## Appendix A. Definitions of the Different Types of Violence

Violence in older adults has various manifestations [11,52]:

- Physical violence: Physical violence involves causing harm to someone’s body (e.g., restraining an older adult against their will or hitting them). It can also include neglect, which is the failure to take necessary actions (e.g., withholding food, medication, or access to healthcare). Physical violence can result in bodily injury, severe wounds, permanent disabilities, and even death.
- Psychological violence: Psychological violence includes behavior that can cause emotional pain, fear, and harm to a person’s mental health (e.g., infantilizing, insulting, and isolating the older adult). It also includes neglecting the emotional and social needs of the older adult.
- Economic violence: Economic violence occurs when behavior is exhibited that causes a person financial harm (e.g., forging checks, forcing older adults to hand over their pension, or pressuring them to change names on their will).
- Socio-economic violence: This form of violence is associated with factors such as gender, sexual orientation, legal status, disability, or age. It involves discrimination and/or the denial of opportunities, assistance, and services to older adults, such as refusing to rent to them or denying access to medication they are entitled to.
- Sexual violence: According to the World Health Organization (WHO), sexual violence refers to “Any sexual act that is perpetrated against someone’s will, committed by any person regardless of their relationship to the victim, in any setting”. It includes, but is not limited to, rape, attempted rape, and sexual slavery, as well as unwanted touching, threatened sexual violence, verbal sexual harassment, and sexual neglect.

## Appendix B. Development of the Comprehensive Guidance to Identify and Care for Older Adults Affected by Violence

The development of the draft guidance was performed through an iterative process of literature reviews, input from national and international experts, and focus group discussions before piloting was conducted.

From July–August 2023, a search was conducted in PubMed and Google Scholar for literature and guidelines on violence against older adults (such as elder abuse) and sexual violence (preferably involving older adults). MeSH terms, combined with keywords identified in previous reviews, were used to develop the search strategy. Due to limited literature and the absence of guidelines on sexual violence in this population, the scope was expanded to include a broader group, with relevant information manually selected. Predefined criteria excluded non-Dutch, French, or English texts, abstracts, case reports, inaccessible articles, and guidelines not aimed at healthcare providers. No publication date restrictions were applied. The guidelines were analyzed using thematic analysis in NVivo12 (QSR International). The literature review provided valuable insights into current evidence-based best practices. Key themes identified in the review included: (1) *the importance of applying trauma-informed principles and how to implement them, (2) the rationale for incorporating routine screening as a standard of care, (3) the need for institutional guidance and its components, and (4) the necessity of holistic care and its implications*. Additionally, the review revealed a significant gap in the existing literature and guidelines, with no screening tools specifically addressing sexual violence in older adults, nor clear care and referral pathways for this population.

The gaps identified in the literature review led to the organization of focus groups between September and October 2024. Participants, recruited from July to October 2024, were Dutch- or French-speaking healthcare providers with experience in working with older adults affected by violence. In addition to hospital settings, residential care settings were included due to their extensive experience in screening older adults (e.g., BelRAI). A variety of recruitment strategies were used, including outreach through newsletters, professional associations, advocacy groups, government agencies, and snowball sampling. To ensure the quality of both the process and outcomes, several strategies were implemented, such as method triangulation, external audits, and detailed descriptions of methods and findings. Written consent was obtained from all participants prior to each focus group. In total, 17 healthcare providers participated in the study, with a mean age of 46 years, the majority being female (*N* = 14), and working in a hospital setting (*N* = 15). Disciplines varied between participants: physicians (*N* = 3), nurses (*N* = 6), coordinators of Sexual Assault Centers (*N* = 2), occupational therapists (*N* = 2), psychologists (*N* = 2), a social worker (*N* = 1), and a nursing assistant (*N* = 1). Thematic analysis of the transcripts in NVivo12 (QSR International) revealed six key themes: (1) *routine screening: a utopia?, (2) the balancing act in finding the right screening protocol, (3) the essential need for training and education, (4) respecting the patient’s autonomy in providing holistic care, (5) overcoming barriers to healthcare access and provision, and (6) keeping the future in mind: sustainability as a driving force.*

Stakeholder input was sought multiple times throughout the research process, beginning with the early stages (e.g., development of research questions) and continuing through to the final analysis (e.g., data from the pilot study). Their feedback shaped the research approach, enhanced the accuracy, and improved the overall quality of the guidance (e.g., identifying key areas of focus, selecting appropriate research methods, and addressing potential challenges). The stakeholders involved included a steering committee of ten Belgian experts specializing in elderly care, (sexual) violence in older adults, and sexuality in older adults; an international expert in elder abuse and senior-friendly environments, and a social worker who previously worked in a geriatric department and is currently employed as a staff and policy advisor within a hospital.

## Appendix C. Pilot Version of the Screening Protocol

For the pilot study, a one-time or dual screening protocol was developed, informed by both the literature review, with validated screening tools, and insights from focus groups. The protocol is designed to guide healthcare providers in incorporating the screening questions naturally during the intake or anamnesis.

Screening is only conducted when the older adult is alone, without the presence of (potentially abusive) relatives. While a suggested sequence of questions is provided, the protocol is flexible. Healthcare providers are encouraged to adapt the order based on the clinical context. Ideally, questions related to violence should be asked towards the end of the conversation, once a basic level of trust has been established with the older adult.

Older adults with cognitive impairments cannot participate in the pilot study. The cognitive abilities can be assessed by using a questionnaire, such as the Mini-Mental State Examination (MMSE), or through some informal questions, such as the following:

- What is your name? - What is your date of birth? - How old are you currently? - What year is it? - What month is it? - What day is it? - Who is the King of Belgium?	
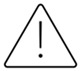

I. First Screening

The following questions, which can be used flexibly, are recommended for asking older adults:

- Can you indicate how you are feeling today?
  ▪ What makes you feel this way?
- Where do you live?
- Do you live alone or with someone?
  ▪ If you live with someone, can you tell me who they are?
- What do you think about your living situation?
  ▪ If you could change anything about your current living situation, what would it be?
  ▪ Do you feel safe there?
    - If not, what makes you feel unsafe?
- Does anyone take care of you?
  ▪ If yes, how would you describe your relationship with this person?
  ▪ If yes, how do you feel about the care you receive?
  ▪ If yes, do you feel safe with this person or these people?
    - If not, what makes you feel unsafe with this person/these people?

We recommend healthcare providers ask the following follow-up questions when there are suspicions or disclosures of violence:

▪ When did [the incident] occur?
  ▪ Have you experienced this within the last 72 h, the past week, the past month, or more than a month ago?
  ▪ How often did [the incident] happen?
▪ What would help you feel safer?/What changes would you like to see?
▪ What do you expect from me?
▪ To what extent would you like to receive additional care? What type of care would you prefer?

II. Second screening

- Can you indicate how you are feeling today?
  ▪ What makes you feel this way?
- Why are you currently in the hospital?
  ▪ For fractures and injuries, ask what happened. These injuries may possibly be the result of violence (for example: a fall because of being pushed, bedsores from neglect, etc.
- Can you describe your social network?
  ▪ How do you experience your interactions with your social network?
- Who do you have contact with on a weekly basis?
  ▪ How do you experience these interactions?
- Are there sometimes tensions in your contacts with your network?
  ▪ If yes, can you give an example?
- Do you ever feel afraid of someone in your social network?
  ▪ If yes, what makes you afraid of this person?
- Is there someone you can turn to when you have problems?
  ▪ If so, who is this person?
- [If not yet answered] Do you still have a partner?
  ▪ How would you describe your intimate life? Do you still hug each other? Do you still touch each other? Is there still sexual contact?
  ▪ How satisfied are you with your intimate life?
  ▪ If not satisfied, what would you like to change?
- Is there someone you are currently taking care of?
  ▪ If yes, what kind of care do you provide?
  ▪ Do you sometimes experience tensions in this caregiving relationship? Can you give an example?
- Is someone currently taking care of you?
  ▪ Who is providing care for you right now?
  ▪ How do you experience this care?
  ▪ Do you sometimes experience tensions in this caregiving relationship? Can you give an example?
- Do you feel safe in your current living situation?
  ▪ If not, what makes you feel unsafe?
- Have you ever felt unsafe in your living situation?
  ▪ If yes, what made you feel unsafe?

When signs of violence are present, it is crucial for healthcare providers to identify both the type of violence experienced and the time that has passed since the incident. This information is essential for determining the appropriate care pathway. The screening questions were developed using insights from the literature, focus groups, and existing validated tools, including behavior-specific questions from the UN-MENAMAIS (Understanding the Mechanisms, Nature, Magnitude and Impact of Sexual Violence in Belgium study) and the Elder Abuse Suspicion Index (EASI). To enhance clarity, compound questions from the EASI were broken down into separate items to avoid multiple prompts within a single sentence. Since the EASI does not directly address sexual violence, behavior-specific questions from the UN-MENAMAIS study were added. This process led to the following screening questions exploring the type of violence:

“I’m going to ask you a few yes or no questions to help identify any actions that may have violated your rights.”

- Has anyone ever prevented you from buying things like food, medication, glasses, or a hearing aid?
- Has anyone ever stopped you from seeking medical help?
- Has anyone ever prevented you from meeting people you wanted to see?
- Have you ever been forced to sign documents against your will?
- Has anyone ever used your money without your permission?
- Have you ever felt ashamed because of something someone said to you?
- Have you ever felt threatened by someone?
- Has anyone ever caused you physical harm?
- Has anyone ever partially or fully undressed you, or have you ever had to undress yourself against your will?
- Have you ever been forced to kiss someone, or has someone ever kissed you against your will?
- Has anyone ever touched you in intimate areas against your will?
- Have you ever been compelled to engage in oral, vaginal, or anal sex against your will?

We also recommend the following follow-up questions in case of suspicions or disclosures of violence:

- When did [the incident] occur?
- Have you experienced this within the last 72 h, the past week, the past month, or more than a month ago?
- How often did [the incident] happen?
- What would help you feel safer?/What changes would you like to see?
- What do you expect from me?
- To what extent would you like to receive additional care? What type of care would you prefer?

## Appendix D. Evaluation Questionnaire Completed by Healthcare Providers (in Dutch and French)

**Dutch**

- In welke mate vind je het screeningsprotocol een goed instrument om geweldervaringen bij je patiënten op te sporen? [5: zeer goed instrument–1: slecht instrument]
  ▪ Indien 2 (matig instrument) of 1 (slecht instrument): Hoe kan dit volgens jou beter?
- In welke mate zou je het gebruik van het screeningsprotocol aan collega’s aanbevelen? [5: sterk aanbevelen–1: niet aanbevelen]
  ▪ Indien 2 (eerder niet aanbevelen) of 1 (niet aanbevelen): Kan je meer duiding geven waarom je het gebruik niet aanbeveelt?
- Hoe gemakkelijk vond je het om het screeningsprotocol in te bedden in het geriatrisch assessment? [5: zeer makkelijk–1: zeer moeilijk]
  ▪ Indien 2 (eerder moeilijk) of 1 (zeer moeilijk): Hoe kunnen we het integreren van het screeningsprotocol in het geriatrisch assessment makkelijker maken?
- In welke mate vind je dat het afnemen van het screeningsprotocol een inspanning van jou vroeg? [5: geen inspanning–1: zeer veel inspanning]
  ▪ Indien 2 (veel inspanning) of 1 (zeer veel inspanning): Wat maakt dat dit van jou een inspanning vroeg?
- In welke mate verstoorde het screeningsprotocol jouw andere prioriteiten in de zorg aan de patient? [5: helemaal geen verstoring–1: grote verstoring]
  ▪ Indien 2 (eerder wel een verstoring) of 1 (grote verstoring): Hoe kunnen we ervoor zorgen dat het screeningsprotocol minder conflicteert met andere prioriteiten?
- Indien je een patiënt had met een geweldervaring, hebben de zorg- en doorverwijzingstrajecten jou goed ondersteund bij het bieden van gepaste zorg? [5: zeer goed ondersteund–1: helemaal niet ondersteund]
  ▪ Indien 2 (weinig ondersteund) of 1 (helemaal niet ondersteund): Hoe kan dit volgens jou beter?
- In welke mate denk je dat de leidraad (screeningprotocol en zorg- en doorverwijzingstrajecten) positief bijdragen heeft aan het welzijn van je patiënt? [5: heel positieve bijdrage–1: heel negatieve bijdrage]
  ▪ Indien 2 (negatieve bijdrage) of 1 (heel negatieve bijdrage): Hoe kunnen we via de leidraad wel positief bijdragen aan het welzijn van je patiënt?
- Welke voordelen denk je dat het gebruik van de richtlijn (het screeningsprotocol en de zorg- en doorverwijzingstrajecten) heeft?
- Welke nadelen denk je dat het gebruik van de richtlijn (het screeningsprotocol en de zorg- en doorverwijzingstrajecten) heeft?
- In welke mate denk je dat patiënten het aanvaardbaar vonden om de screeningsvragen te ontvangen tijdens het geriatrisch assessment? [5: zeer aanvaardbaar–1: zeer onaanvaardbaar]
  ▪ Indien 2 (eerder onaanvaardbaar) of 1 (zeer onaanvaardbaar): Kan je verduidelijken wat je patiënten onaanvaardbaar vonden?
- In welke mate vonden je patiënten volgens jou de inhoud van de screeningsvragen aanvaardbaar? [5: zeer aanvaardbaar–1: zeer onaanvaardbaar]
  ▪ Indien 2 (eerder onaanvaardbaar) of 1 (zeer onaanvaardbaar): Kan je verduidelijken wat je patiënten onaanvaardbaar vonden?
- In welke mate vonden je patiënten volgens jou de formulering van de screeningsvragen aanvaardbaar? [5: zeer aanvaardbaar–1: zeer onaanvaardbaar]
  ▪ Indien 2 (eerder onaanvaardbaar) of 1 (zeer onaanvaardbaar): Kan je verduidelijken wat je patiënten onaanvaardbaar vonden?
- Welke suggesties ter verbetering heb je rond de inhoud van de screeningsvragen?
- Welke suggesties ter verbetering heb je rond de formulering van de screeningsvragen?
- Hoe veel vertrouwen had je in jezelf bij het gebruiken van het screeningsprotocol? [5: veel vertrouwen–1: helemaal geen vertrouwen]
  ▪ Indien 2 (eerder geen vertrouwen) of 1 (helemaal geen vertrouwen): Hoe kunnen we ervoor zorgen dat je meer vertrouwen hebt in jezelf bij het afnemen van het screeningsprotocol?
- Welke zaken in de richtlijn hebben meer verduidelijking nodig volgens jou?
- In welke mate heb je het gevoel dat het screeningsprotocol jou voldoende kennis en vaardigheden aanleverde om aan de slag te gaan? [5: veel kennis en vaardigheden bijgebracht–1: geen kennis en vaardigheden bijgebracht]
  ▪ Indien 2 (weinig kennis en vaardigheden bijgebracht) of 1 (geen kennis en vaardigheden bijgebracht): Welke kennis en vaardigheden zouden volgens jou bijgespijkerd moeten worden om de screening op geweld bij je patiënten af te nemen?
- Indien je een patiënt had met een geweldervaring, heb je het gevoel dat de zorg- en doorverwijzingstrajecten jou voldoende kennis en vaardigheden aanleverde om aan de slag te gaan? [5: veel kennis en vaardigheden bijgebracht–1: geen kennis en vaardigheden bijgebracht]
  ▪ Indien 2 (weinig kennis en vaardigheden bijgebracht) of 1 (geen kennis en vaardigheden bijgebracht): Welke kennis en vaardigheden zouden volgens jou bijgespijkerd moeten worden om je patiënten met geweldervaringen gepast door te verwijzen?
- Welke ervaringen rond de pilootstudie wil je nog met ons delen?

**French**

- Dans quelle mesure estimez-vous que le protocole de dépistage est un bon outil pour détecter des violences subies par vos patient·e·s? [5: très bon outil—1: mauvais outil]
  ▪ Quand 2 (outil moyen) ou 1 (mauvais outil): Comment pourrait-on l’améliorer, selon vous?
- Dans quelle mesure recommanderiez-vous le recours au protocole de dépistage à des collègues? [5: vivement recommandé–1: pas recommandé]
  ▪ Quand 2 (peu recommandé) ou 1 (pas recommandé): Pouvez-vous expliquer pourquoi vous ne le recommanderiez pas?
- Dans quelle mesure avez-vous trouvé facile d’intégrer le protocole de dépistage dans l’évaluation gériatrique? [5: très facile–1: très difficile]
  ▪ Quand 2 (plutôt difficile) ou 1 (très difficile): Comment pourrions-nous rendre l’intégration du protocole de dépistage dans l’évaluation gériatrique plus facile?
- Dans quelle mesure trouvez-vous que le fait de suivre le protocole de dépistage vous a demandé un effort? [5: aucun effort–1: effort considérable]
  ▪ Quand 2 (effort importante) ou 1 (effort considérable): Qu’est-ce qui vous a demandé un effort?
- Dans quelle mesure le protocole de dépistage a-t-il interféré avec vos autres priorités en matière de soins aux patient·e·s? [5: aucune interférence–1: interférence majeure]
  ▪ Quand 2 (une certaine interférence) ou 1 (interférence majeure): Comment faire en sorte que le protocole de dépistage soit moins en conflit avec d’autres priorités?
- Si vous avez soigné un·e patient·e victime de violences, les filières de prise en charge et d’orientation vous ont-elles été utiles pour prodiguer les soins appropriés? [5: très utile–1: pas du tout utiles]
  ▪ Quand 2 (peu utiles) ou 1 (pas du tout utiles): Comment pourrait-on les améliorer, selon vous?
- Dans quelle mesure pensez-vous que le guide (dépistage et filières de prise en charge et d’orientation) a contribué de manière positive au bien-être de vos patient·e·s? [5: contribution très positive–1: contribution très négative]
  ▪ Quand 2 (contribution négative) ou 1 (contribution très négative): Comment apporter une contribution positive au bien-être de vos patient·e·s?
- Selon vous, quels sont les avantages liés à l’utilisation du guide (le protocole de dépistage et les filières de prise en charge et d’orientation)?
- Selon vous, quels sont les inconvénients liés à l’utilisation du guide (le protocole de dépistage et les filières de prise en charge et d’orientation)?
- Dans quelle mesure pensez-vous que vos patient·e·s ont trouvé acceptable de recevoir les questions de dépistage lors de l’évaluation gériatrique? [5: très acceptable–1: totalement inacceptable]
  ▪ Quand 2 (plutôt inacceptable) ou 1 (totalement inacceptable): Pouvez-vous préciser ce que votre patient·e a jugé inacceptable?
- Dans quelle mesure pensez-vous que vos patient·e·s ont trouvé le contenu des questions de dépistage acceptable? [5: très acceptable–1: totalement inacceptable]
  ▪ Quand 2 (plutôt inacceptable) ou 1 (totalement inacceptable): Pouvez-vous préciser ce que vos patient·e·s ont jugé inacceptable?
- Dans quelle mesure pensez-vous que vos patient·e·s ont trouvé la formulation des questions de dépistage acceptable? [5: très acceptable–1: totalement inacceptable]
  ▪ Quand 2 (plutôt inacceptable) ou 1 (totalement inacceptable): Pouvez-vous préciser ce que vos patient·e·s ont jugé inacceptable?
- Avez-vous des suggestions pour améliorer le contenu des questions de dépistage?
- Avez-vous des suggestions pour améliorer la formulation des questions de dépistage?
- Quel a été votre niveau de confiance en vous lorsque vous avez utilisé le protocole de dépistage? [5: une confiance élevée–1: pas du tout de confiance]
  ▪ Quand 2 (pas beaucoup confiance) ou 1 (pas du tout de confiance): Comment pouvons-nous faire en sorte que vous ayez davantage confiance en vous lorsque vous utilisez le protocole de dépistage?
- Quels sont les éléments du guide qui, selon vous, méritent d’être clarifiés?
- Dans quelle mesure pensez-vous que le protocole de dépistage vous a permis d’acquérir des connaissances et des compétences suffisantes pour travailler? [5: de nombreuses connaissances et compétences–1: aucunes connaissances et compétences]
  ▪ Quand 2 (peu connaissances et compétences) ou 1 (aucunes connaissances et compétences): Selon vous, quelles connaissances et compétences devraient être mises à jour pour effectuer un dépistage des violences chez vos patient·e·s?
- Si vous avez dû soigner un·e patient·e victime de violences, dans quelle mesure pensez-vous que les filières de prise en charge et d’orientation vous ont permis d’acquérir des connaissances et des compétences suffisantes pour travailler? [5: de nombreuses connaissances et compétences–1: aucunes connaissances et compétences]
  ▪ Quand 2 (peu connaissances et compétences) ou 1 (aucunes connaissances et compétences): Selon vous, quelles connaissances et compétences devraient être mises à jour pour orienter efficacement vos patient·e·s ayant subi des violences?
- De quelles expériences liées à l’étude pilote souhaiteriez-vous encore nous faire part?

## Appendix E. Final Version of the Screening Protocol

Rather than using a one-time or dual screening approach, we propose a more flexible screening protocol that includes both broad and behaviorally specific questions, asked in a conversational, interview-style format. This approach creates space for openness and may encourage disclosure, especially in cases where older adults do not label themselves as victims or do not recognize their experiences as violent. For this method to be effective, it is crucial that healthcare providers receive adequate training and education on violence in older adults. Therefore, they are able to recognize potential signs of abuse and ask relevant follow-up questions. This approach also requires that providers have enough time and strong communication skills, such as attentive listening and the ability to ask sensitive, probing questions in a respectful and supportive manner, which may also require additional educational opportunities. Preferably, questions about violence should be integrated towards the end of the geriatric assessment or intake conversation, as this timing helps foster trust and strengthen the relationship between healthcare providers and patients. It is important to allocate sufficient time for the conversation and communicate this clearly to the patient. This helps reduce the pressure of feeling like a burden and emphasizes that the healthcare provider values listening to them. Due to its time-intensiveness, it may be necessary to have multiple conversations with the older adult.

You can start the conversation by asking a general, open-ended question:

*“Which experiences have shaped your life the most?”*

This type of question enables a natural conversation and allows the older adult to speak freely. It may also naturally lead to information indicating potential sources of concern, tension, conflict, or violence, both by known and unknown perpetrators. If patients tell about their social network, you can use the following follow-up questions:

*“Have you ever felt unsafe in your living situation?”*

*“Are there sometimes conflicts with those around you?”*

*“If yes, can you tell me more?”/“If yes, can you give an example?”*

*“Have you ever felt afraid of someone?*

*“If yes, what makes you afraid of this person?”*

The following behaviorally specific questions can also be naturally integrated into the conversation to help identify the presence and nature of violence experienced. These questions avoid using labels like “abuse” or “violence,” which individuals may not relate to, and instead focus on concrete behaviors or experiences of violence or transgressive behavior. You can introduce the questions in the following manner:

*“I would like to ask you some questions to explore whether you’ve ever experienced transgressive or violent situations, either now or in the past. I ask these questions to everyone, because many older adults experience some form of transgressive behaviors or violence, often by people they know well. If you’re not comfortable, you don’t have to answer every question. Is that okay with you?”*

- *“Has anyone ever prevented you from buying things like food, medication, glasses, or a hearing aid?”*
- *“Has anyone ever stopped you from seeking medical help?”*
- *“Has anyone ever prevented you from meeting people you wanted to see?”*
- *“Have you ever been forced to sign documents against your will?”*
- *“Has anyone ever used your money without your permission?”*
- *“Have you ever felt ashamed because of something someone said to you?”*
- *“Have you ever felt threatened by someone?”*
- *“Has anyone ever caused you physical harm?”*
- *“Has anyone ever partially or fully undressed you, or have you ever had to undress yourself against your will?”*
- *“Have you ever been forced to kiss someone, or has someone ever kissed you against your will?”*
- *“Has anyone ever touched you in intimate areas against your will?”*
- *“Have you ever been compelled to engage in oral, vaginal, or anal sex against your will?”*

If the screening results in a positive screening or disclosure. It is to ask follow-up questions. You can introduce these questions as follows:

*“To make sure I care for you in the most appropriate way, I am going to ask a few additional questions if that is ok?”*

We suggest the following follow-up questions:

- *“When did [the incident] occur?”*
- *“Have you experienced this within the past week, the past month, or more than a month ago?”*
- *“How often did [the incident] happen?”*
- *“What would help you feel safer?”/“What changes would you like to see?”*
- *“How can I be of help in making these changes happen?”*
- *“To what extent would you like to receive additional care?”*
- *“What type of care would you prefer?”*
